# Editorial: Studies on life at the energetic edge – from laboratory experiments to field-based investigations, volume II

**DOI:** 10.3389/fmicb.2023.1351761

**Published:** 2024-01-04

**Authors:** Tori M. Hoehler, Jan P. Amend, Bo Barker Jørgensen, Victoria J. Orphan, Mark A. Lever

**Affiliations:** ^1^Exobiology Branch, NASA Ames Research Center, Moffett Field, CA, United States; ^2^Department of Earth Sciences and Department of Biological Sciences, University of Southern California, Los Angeles, CA, United States; ^3^Department of Biology, Aarhus University, Aarhus, Denmark; ^4^Department of Geological and Planetary Sciences, California Institute of Technology, Pasadena, CA, United States; ^5^Marine Science Institute, The University of Texas at Austin, Austin, TX, United States

**Keywords:** energy limitation, deep biosphere, aquatic sediments, cryopeg, serpentinizing systems, hydrothermal systems

Among the diverse inhabitants of Earth's biosphere, microorganisms reign supreme in their capabilities to occupy an expansive range of ecological niches. We often conceive of the fringes of our biosphere in terms of physicochemical “extremes”, but perhaps the most pervasive environmental challenge to microbial life is that of extreme energy limitation (Hoehler and Jørgensen, 2013; Lever et al., 2015; Bradley et al., 2020). Globally, for example, the marine deep biosphere occupies a volume and hosts a biomass comparable to that of the overlying oceans, yet that deep biosphere is sustained by an energy flux about 1,000 times lower (Hoehler et al., 2023). Both in the sub-seafloor and in the continental subsurface, these energy-starved organisms exist at the interface between the inhabited and uninhabited realms of our planet. To understand their physiology in the face of extreme energy limitation would, therefore, be to understand the capabilities and limitations of the ultimate arbiters of chemical exchange between the biosphere and the geosphere.

Our awareness and understanding of life at the energetic edge has grown considerably over the last few decades, yet much remains to be learned. What factors determine the minimal energy requirements of microorganisms, and how do those requirements vary with the physicochemical environment? What physiological traits promote survival under extreme energy limitation? Do those traits evolve as a specific adaptation to energy limitation, or are they a fortuitous result of adaptation to other conditions (exaptation)? Do any physiological differences relating to energy limitation translate to differing capabilities and limitations with respect to biogeochemical processing? Natural ecosystems provide a unique window into populations exposed to energy limitation over long timescales that are intractable in laboratory settings. This special Research Topic focuses on such systems: on advances being made at the frontiers of environmental microbiology.

Jaussi et al. quantified cell-specific catabolic energy yields in several marine sediments, for both the total cell population and for sulfate-reducing bacteria (SRB), specifically. They observed that cell-specific energy yields for the total population diminished over orders of magnitude with increasing sediment depth (age), but yields for SRB specifically appeared to reach a stable minimum much higher than that of the overall population. Taxonomic or metabolic differences thus appear to factor significantly into minimum energy requirements. This study leveraged sensitive radiotracer techniques to measure metabolic process rates. For those interested in pursuing similar techniques, Schubert and Kallmeyer have offered a deep dive into the methodology and optimization of liquid scintillation counting (LSC)—a “how-to” guide for pushing the sensitivity of radiotracer methods to the levels required for characterizing life at the energetic edge. Kanaan et al. modeled the energy requirements of microbes entrapped in a “cryopeg”—a volume of brine isolated within permafrost for 40,000 years. While subzero temperatures could potentially depress microbial energy requirements, the findings instead suggest that the need to produce extracellular enzymes and cryo- and osmoprotectant compounds results in an average per-cell requirement comparable to that observed by Jaussi et al. for SRB and well above that observed for non-SRB in cold marine sediments.

Two studies focused on microbial adaptations in serpentinizing systems, where water–rock reactions drive the fluid chemistry toward high pH and highly reducing and extremely CO_2_-poor conditions. Thieringer et al. used metagenome analyses to investigate differences in ecophysiologies among three distinct populations of CO_2_-reducing methanogens. While all were found to share the potential for DNA scavenging, possibly as a strategy to mitigate phosphorus and nitrogen limitation, genomic differences suggest that organisms inhabiting the most alkaline fluids adopt distinctly different strategies for coping with this energetically challenging environment—a niche differentiation that supports their coexistence. Working in the same system, Rempfert et al. performed a comprehensive analysis of membrane-forming intact polar lipids (IPLs). The lipidome, dominated by diether glycolipids, reflects a unique combination of membrane adaptations—including pervasive modifications likely associated with phosphate limitation—to enable microorganisms' survival in these conditions.

Three studies considered underexplored metabolic potential in the environment. Holden and Sistu reviewed the drivers of formate cycling by CO_2_-reducing *Methanococci* and organotrophic *Thermococci*. Both have the genetic potential to produce H_2_ from formate and subsequently catabolize H_2_, but it is not known whether such production is prevalent relative to the direct utilization of environmental H_2_. By considering H_2_ and formate concentrations in hydrothermal fluids and environmental distributions, genomes, and biochemical data on *Methanococci* and *Thermococci*, Holden and Sistu argue that both preferentially utilize H_2_, while formate becomes important primarily under low-H_2_ conditions. Sonke and Trembath-Reichert analyzed published metagenomic and -transcriptomic datasets from a wide range of habitats to investigate the potential importance of oxalate as a microbial energy source. While oxalate is widespread in many environments, both in association with minerals and released by photosynthetic organisms, its typically very low dissolved concentrations suggest rapid turnover. Sonke and Trembath-Reichert demonstrate widespread potential for microbial oxalotrophy, especially in marine environments, where oxalotrophy is thermodynamically favorable and may represent a widely overlooked microbial energy source. Vigderovich et al. used a series of incubation experiments to explore the relationship between iron reduction and aerobic methanotrophy in lake sediments. Rates of methanotrophy and, perhaps surprisingly, accumulation of ferrous iron were both enhanced by the addition of oxygen to hematite-amended incubations. The results suggest a complex interplay between these aerobic and anaerobic microbially mediated processes and a potential role for iron recycling in the survival of aerobic methanotrophs under hypoxic conditions.

Finally, Weeks et al. considered “the edge” for photosynthetic microorganisms. Working in 12 Yellowstone hot springs, they characterized microbial community composition and a suite of physicochemical variables across the “photosynthetic fringe”—the region in which an outflow channel visibly transitions from chemotrophic to highly pigmented photosynthetic microbial communities. While changes in temperature, pH, and sulfide content have been previously proposed to control the location of the transition, such factors only explain about 35% of the variation in community composition, indicating that the microbial community of photosynthetic fringe environments is a more complex interplay of these and other factors.

With this special Research Topic, we invite you to explore these recent advances in understanding life at the energetic edge!

## Author contributions

TH: Writing—original draft. JA: Writing—review & editing. BJ: Writing—review & editing. VO: Writing—review & editing. ML: Writing—original draft, Writing—review & editing.
